# Retrograde Lidocaine Spray for Awake Extubation in Pediatric ENT Surgery: Optimizing Tonsillectomy and Adenoidectomy Outcomes

**DOI:** 10.7759/cureus.68293

**Published:** 2024-08-31

**Authors:** Zachrieh Alhaj, Zaid Almubaid, Omayr Irshad, Gilan Choucair, Sharif Mohamed

**Affiliations:** 1 Medicine, John Sealy School of Medicine, University of Texas Medical Branch, Galveston, USA; 2 Anesthesiology, John Sealy School of Medicine, University of Texas Medical Branch, Galveston, USA; 3 Biology, University of Houston, Houston, USA; 4 Anesthesiology, University of Texas Medical Branch, Galveston, USA

**Keywords:** laryngeal edema, pediatric tonsillectomy and adenoidectomy, otolaryngology, anesthesiology, pediatrics

## Abstract

Laryngeal edema, a frequent manifestation of acute inflammation, is particularly significant due to the potential obstruction of the laryngeal orifice caused by swelling of the epiglottis and vocal cords. This presents as a risk factor that can lead to severe airway obstruction. Traditionally, deep extubation is the preferred form of extubation because it is more comfortable for the patient, eliminates the airway reflexes, and minimizes the risk of laryngeal edema. Difficult mask ventilation (DMV), characterized by an unassisted anesthesiologist's inability to maintain oxygen saturation levels above 92% or to prevent or correct signs of inadequate ventilation during positive-pressure mask ventilation under general anesthesia, necessitates an awake extubation approach. In the following case, combining the need to minimize airway reflexes through a deep extubation with the need for an awake intubation required an alternative method.

Our patient is a 10-year-old male who presented with obstructive sleep apnea and tonsillar hypertrophy. The patient had a history of snoring and difficult intubation (three attempts), classifying him as a DMV risk. However, due to the difficult intubation, there was concern for laryngeal edema following the procedure that would necessitate a deep extubation. To effectively combine the two procedures, a retrograde lidocaine spray was used to numb the airway, which would allow for awake extubation without the associated coughing and bucking.

Deep extubation is a common anesthetic technique used in laryngeal surgeries, but it is often not an option for high-risk patients. For such patients, awake extubation is an alternative. In our case, our patient was at high risk for laryngeal edema. In awake extubation, lidocaine spray is used for minimal coughing and bucking because it numbs the upper airway and allows the patient to tolerate the breathing tube without stimulating the gag reflex. The use of retrograde lidocaine spray for awake extubation in patients at high risk for laryngeal edema presents a promising alternative to traditional methods. This case demonstrates the effectiveness of retrograde lidocaine spray in awake extubation to reduce coughing and bucking by numbing the upper airway in a DMV situation while also avoiding complications in a high-risk patient.

## Introduction

Laryngeal edema, a frequent side effect of airway instrumentation, is particularly significant due to the potential obstruction of the laryngeal airway caused by swelling of the epiglottis, the vocal cords, and the trachea [1]. This presents as a risk factor that can lead to stridor, making it critical to avoid any risk factors that might provoke coughing or airway irritation during extubation. Managing extubation in patients at high risk for laryngeal edema presents a significant clinical challenge. Traditionally, deep extubation is the preferred form of extubation to avoid coughing and bucking because of the minimization of airway reflexes [2]. Additionally, this minimizes the risk of laryngeal edema.

Awake extubation is preferred if there is a history of difficult intubation or difficult mask ventilation (DMV), characterized by an unassisted anesthesiologist's inability to maintain oxygen saturation levels above 92% on pulse oximetry or to prevent or correct signs of inadequate ventilation during positive-pressure mask ventilation under general anesthesia [3]. In the following case, combining the need to minimize airway stimulation through a deep extubation with the need for an awake extubation requires an alternative method.

Retrograde lidocaine spray offers a solution in these scenarios. By numbing the upper airway, this technique can suppress airway reflexes, allowing for a smoother and safer awake extubation [4]. This approach leverages the benefits of awake extubation, maintaining spontaneous ventilation while avoiding the risks associated with deep extubation in patients who are challenging to ventilate.

## Case presentation

Our patient is a 10-year-old male who presented with obstructive sleep apnea and tonsillar hypertrophy. The patient also has a past medical history of pneumothorax and sleep-disordered breathing. He underwent a tonsillectomy and adenoidectomy (T&A) under general anesthesia. The patient had a history of snoring and difficult intubation (three attempts), classifying him as a difficult airway risk; thus, awake extubation was recommended [5]. However, due to the multiple trials of intubation, there was concern for laryngeal edema that could get exacerbated by coughing and bucking during awake extubation. A retrograde lidocaine spray was used to numb the airway to allow for awake extubation without the associated coughing and bucking. The procedure involved suctioning the upper airway and deflating the endotracheal tube (ETT) cuff, connecting the lidocaine syringe to the CO2 sampling port, and increasing the adjustable pressure-limiting (APL) valve to 40 mmH2O to facilitate retrograde flow 'leak' around the endotracheal tube that was loaded with lidocaine to spray the airway retrograde from down upwards [6]. The patient was woken up after two to three minutes, and the tube was pulled out as the patient spontaneously breathed on his own. This allowed for an awake extubation while reducing the coughing, which minimized trauma and the risk of laryngeal edema. 

Following the procedure, our patient was monitored for any signs of respiratory distress. The patient was stable and exhibited regular breathing patterns without signs of laryngeal edema. The postoperative period was uneventful, and the T&A with the use of retrograde lidocaine spray for awake, deep extubation proved to be a safe and effective approach for our patient.

## Discussion

Our patient was a difficult intubation patient, which would require an awake extubation. However, this cannot be done without overlooking the benefits of a deep extubation, primarily the lack of reflexes, such as coughing, which will minimize exacerbating the expected laryngeal edema after a traumatic intubation. The use of the retrograde lidocaine spray allowed for the spontaneous ventilation of awake extubation while also avoiding the risks of awake extubations, such as laryngospasms and coughing, by numbing the airway [7]. 

However, retrograde lidocaine therapy has its risks, such as a potentially delayed extubation, which could result in aspirations [8]. Additionally, because of this concern for aspiration, the patient may not have any oral intake for two hours following extubation, which may be cumbersome in a pediatric population that may want food, water, or popsicles following surgery [8]. Our patient remained stable throughout the procedure and had no complications, demonstrating the safety and efficacy of this approach. 

## Conclusions

The use of retrograde lidocaine spray for awake extubation in patients at high risk for laryngeal edema presents a promising alternative to traditional methods. This case demonstrates the effectiveness of retrograde lidocaine spray in awake extubation to reduce coughing and bucking, numbing the upper airway in a difficult airway situation while also avoiding complications in a high-risk patient. Further research and clinical trials are warranted to assess the risks and contraindications of this technique, as well as its generalizability to other populations. The successful care of our patient highlights an example of the benefit of retrograde lidocaine spray in awake extubation.
